# A role for specific collagen motifs during wound healing and inflammatory response of fibroblasts in the teleost fish gilthead seabream

**DOI:** 10.1016/j.molimm.2010.12.004

**Published:** 2011-03

**Authors:** Patricia Castillo-Briceño, Dominique Bihan, Michael Nilges, Samir Hamaia, José Meseguer, Alfonsa García-Ayala, Richard W. Farndale, Victoriano Mulero

**Affiliations:** a Department of Cell Biology and Histology, University of Murcia, Murcia 30100, Spain; b Department of Biochemistry, University of Cambridge, Cambridge CB2 1QW, United Kingdom; c Department of Structural Biology and Chemistry, Institut Pasteur, 75724 Paris, France

**Keywords:** Adhesion, Collagen motifs, Extracellular matrix, Fibroblasts, Inflammation, Integrin, Interleukin-1β, Cyclooxygenase-2, *Sparus aurata*, Teleost fish, Wound healing

## Abstract

Specific sites and sequences in collagen to which cells can attach, either directly or through protein intermediaries, were identified using Toolkits of 63-amino acid triple-helical peptides and specific shorter GXX′GEX″ motifs, which have different intrinsic affinity for integrins that mediate cell adhesion and migration. We have previously reported that collagen type I (COL-I) was able to prime *in vitro* the respiratory burst and induce a specific set of immune- and extracellular matrix-related molecules in phagocytes of the teleost fish gilthead seabream (*Sparus aurata* L.). It was also suggested that COL-I would provide an intermediate signal during the early inflammatory response in gilthead seabream. Since fibroblasts are highly involved in the initiation of wound repair and regeneration processes, in the present study SAF-1 cells (gilthead seabream fibroblasts) were used to identify the binding motifs in collagen by end-point and real-time cell adhesion assays using the collagen peptides and Toolkits. We identified the collagen motifs involved in the early magnesium-dependent adhesion of these cells. Furthermore, we found that peptides containing the GFOGER and GLOGEN motifs (where O is hydroxyproline) present high affinity for SAF-1 adhesion, expressed as both cell number and surface covering, while in cell suspensions, these motifs were also able to induce the expression of the genes encoding the proinflammatory molecules interleukin-1β and cyclooxygenase-2. These data suggest that specific collagen motifs are involved in the regulation of the inflammatory and healing responses of teleost fish.

## 1 Introduction

Collagens are major components of the extracellular matrix (ECM) of all metazoans (Kramer, 1994). Fibrillar collagen types I and II are the most abundant proteins of ECM (Wess, 2005), and like the other fibril-forming collagens (types III, V, XI, XXIV and XXVII), they are able to form continuous triple-helical domains (Heino et al., 2009). These collagens can form stable large fibrils and complex fibrous superstructures that are responsible for the tensile strength of the tissues (Perumal et al., 2008; Heino et al., 2009; Herr and Farndale, 2009).

In addition to their structural role, collagens are able to modulate cellular inflammatory response and activities, depending on the microenvironment and the physiological processes involved. Cellular recognition of collagens is mediated by a variety of surface receptors, such as integrins, discoidin domain receptors, and immune related receptors. Upon collagen binding, these receptors may activate other molecules related to remodeling, inflammation and wound healing processes, such as matrix metalloproteases (MMPs), cytokines and growth factors (Vogel, 2001; Tran et al., 2004; Lee et al., 2007; Heino et al., 2009; Schultz and Wysocki, 2009; Boilard et al., 2010). Collagen type I (COL-I) fragments increase the release and activity of interleukin-1β (IL-1β) in human peripheral blood- and adherent-monocytes after attachment in a response mediated by integrin α2β1 (ITGA2B1) and as part of remodeling mechanisms (Pacifici et al., 1991). We also showed in the teleost fish gilthead seabream (*Sparus aurata* L.) that native COL-I can act as a damage associated molecular pattern (DAMP) by increasing the respiratory burst in leukocytes and the mRNA levels of gene coding for IL-1β and other pro-inflammatory molecules in professional phagocytes (Castillo-Briceño et al., 2009).

Particular interest in the role of collagens during wound healing and regeneration arose since collagen membrane devices have been found useful to guide these processes, in which type, origin and processing of collagen may result in differential cellular behavior (Behring et al., 2008). Such studies have been performed with several cell types, but especially with fibroblasts, which are directly relevant to different aspects of tissue engineering (Steffensen et al., 2001; Puklin-Faucher and Sheetz, 2009; Dobaczewski et al., 2010). Modulation of fibroblast behavior by ECM molecules such as the collagens is considered to be triggered by both mechanical and biochemical stimulation (Daley et al., 2008; Farahani and Kloth, 2008; Gjorevski and Nelson, 2009; Jiang et al., 2007). For example, it is known that in mice, fibroblast phenotype and gene expression are altered by their adhesion state (Dhawan et al., 1991), and human fibroblast interaction with a collagenous surface modulates their attachment, morphology, proliferation rate and migration (Rothamel et al., 2004; Behring et al., 2008).

Integrins have been widely studied as mediators of cell binding to collagens and other ECM molecules, especially in early adhesion mechanisms (Knight et al., 2000; Dobler et al., 2006; Puklin-Faucher and Sheetz, 2009). Such binding can be involved in specific cellular processes such as platelet aggregation (Santoro and Cunningham, 1980) or fibroblast adhesion and migration (Behring et al., 2008). It is known that the subset of integrin β1 (ITGB1) heterodimers (α1β1, α2β1, α10β1, α11β1) that are able to bind collagen directly does so through the inserted I-domain (also called A-domain) of the integrin α subunits (Takada et al., 2007), similar to the related structures found in all integrin β subunits (Xiong and Zhang, 2001; Isaji et al., 2009). The affinity of integrins for collagen depends on the accessibility within collagens (Herr and Farndale, 2009) and intrinsic activity of binding sites, the high affinity GXX′GEX″ motifs (where X tends to be hydrophobic, X′ is usually O, and X″ usually R) (Knight et al., 2000; Siljander et al., 2004; Raynal et al., 2006). Despite the strong relevance of collagen–integrin interactions to many aspects of cell biology, integrins are barely characterized at structural or functional level in lower vertebrates.

Toolkits of collagen-derived peptides, overlapping sets of triple-helical, homotrimeric peptides encompassing the entire triple-helical domains of COL II and III, and other specific motifs, have proved useful for studying the specific collagen sequences involved in receptor recognition and downstream cellular responses (Lisman et al., 2006; Raynal et al., 2006; Farndale et al., 2008). In the present study, COL-II- and COL-III-derived peptides were used, for two reasons. Firstly, COL-II and COL-III are phylogenetically close to COL-I and contained in the same clade (Heino et al., 2009), and they are highly conserved between teleost fish and human (for example 88% amino acid similarity between seabream and human collagen I alpha 1). Secondly, the synthesis of many long homotrimeric peptides is far simpler than the heterotrimers that would be required to form a COL-I Toolkit. These peptides have allowed us to identify specific collagen sequences and motifs that directly induce the early gilthead seabream fibroblast response related to adhesion affinity and the synthesis of inflammation-related molecules.

## 2 Materials and methods

### 2.1 Cell culture and materials

The established cell line of caudal fin fibroblasts from *S. aurata* (SAF-1) were purchased in the European Collection of Cell Cultures (ECACC, UK) and cultured at 25 °C in Leibovitz's L-15 media supplemented with 5% FBS, 100 IU/ml penicillin and 100 μg/ml streptomycin (Sigma). The COL-II and-III Toolkits of triple helical peptides, GXX′GEX″ specific motifs, GPP_10_ and CRP (GPO_10,_ where O is hydroxyproline) peptides were synthesized and purified, and their triple helical properties were tested as previously described (Raynal et al., 2006; Smethurst et al., 2007). Also used were the commercial RGD tripeptide, echistatin (a RGD-containing disintegrin), endostatin (carboxyl-terminal domain of collagen XVIII), bovine COL-I (native and denatured at 60 °C during 0.5–3 h), human COL-II, human COL-III and BSA (Sigma), all of them cell culture tested.

### 2.2 End-point cell adhesion assays

Adhesion assays were performed to evaluate the early attachment of fish fibroblasts to different collagen-derived peptides, motifs and related sequences. Plastic 96 well plates for ELISA (Immulon 2 and Nunc) were coated in triplicate with 100 μl per well of the collagens, Toolkit peptides, GXX′GEX″ motifs, GPP_10_ and CRP peptides at 10 μg/ml in 0.01 M acetic acid for at least 16 h at 4 °C based on previous studies (Smethurst et al., 2007). The same process was applied to test RGD triplet, echistatin and endostatin (0.5–50 μg/ml). Then, plates were blocked with 5% BSA in TBS (50 mM Tris–HCl, 140 mM NaCl, pH 7.4) for 1 h at 20 °C and washed to add the cell suspension. Cells were detached following the ECACC specifications, resuspended in 0.1% BSA in TBS and incubated at 1 × 10^5^ cells per well for 50 min at 20 °C, in the presence of 5 mM Mg^2+^ or 5 mM EDTA to enhance or to inhibit, respectively, integrin extracellular domain binding (Raynal et al., 2006). Thereafter, plates were washed, and adherent cells were incubated for 1 h with lysis buffer (0.07 M tri-sodium citrate, 0.3 M citric acid, 0.1% Triton X-100) to estimate the proportion of attached cells from their LDH content, using the LDH cytotoxicity kit (Roche) in a FLUOstar luminometer (BMG LabTechnologies).

### 2.3 Real time cell adhesion (RTCA) assays

The method relies on the increase in electrical impedance of an electrode array coated with adhesive substrates, such as collagen-derived peptides, and the increase in impedance as either cells contact the surface or spread over the surface is reported as a dimensionless parameter termed Cell Index. RTCA plates (Roche) were coated and blocked as in the end-point adhesion assays, but for 1 h each step and at 20 °C. Next, cells in suspension were added with Mg^2+^ or EDTA, or with Mg^2+^ and 50 μg/ml genomic DNA of *Vibrio anguillarum* (VaDNA), as a pathogen associated molecular pattern (PAMP), and left to incubate at 23 °C. The Cell Index was continuously read in a RTCA analyzer (xCELLigence, Roche), with measurements taken every 5 min during the first 3 h, then every 20 min for another 13 h at least.

### 2.4 Modeling analysis of the I-like domain of gilthead seabream ITGB1a in complex with collagen motifs

The ITGB1a I-like domain was modeled on the basis of its sequence similarity with the extracellular domain of ITGB3 heterodimers (1U8C, ITXV) (Xiao et al., 2004; Xiong et al., 2004), using BLAST (NCBI) to search for homologous domains in the PDB (Berman et al., 2000), T-Coffee (Notredame et al., 2000) to align the sequences and Modeller (Eswar et al., 2006) for the actual modeling. This was performed in an automated way with the BisKit molecular modeling Toolkit (Grünberg et al., 2007). Models of the GFOGER, GLOGEN and CRP motifs were obtained by modifying side-chains in the collagen part of the ITGA2 I-domain in complex with the GFOGER motif (PDB code 1DZI) (Emsley et al., 2000), using ad hoc scripts developed for CNS (Brunger et al., 1998). The model of a complex between the ITGB1a I-like domain and the GFOGER, GLOGEN and CRP were obtained by structure superposition of the ITGB1a I-like domain onto the ITGA2 I-domain in the complex with the GFOGER motif. The models were energy minimized and refined in a 12 Å layer of explicit TIP3P solvent model (Jorgensen et al., 1983) with CNS, with the CHARMM Param19 force field (Brooks et al., 1983). The electrostatic analysis was performed using the ACE generalized Born model implemented in CNS (Calimet et al., 2001). The difference in energy was estimated as the difference between the electrostatic, van der Waals and generalized Born contributions to the total energy calculated in the complex and the two molecules separated by 100 Å. We used 4 for the internal dielectric and 80 for the external dielectric constants.

### 2.5 Analysis of gene expression

Fish fibroblasts were seeded in TBS supplemented with 5 mM Mg^2+^ at 1 × 10^6^ cells in 25 cm^2^ flasks which were coated as described for the End-point cell-adhesion assays with collagen-derived peptides or only BSA. GFOGER and GLOGEN specific motifs were also tested in suspension (10 μg/ml) in BSA coated flasks. Then, cells were incubated for 50 min and collected, separating the adhered and the non-adhered fractions to be stored in TRIzol at −80 °C until processing. RNA extraction and treatment with DNase I (1 U/μg RNA) Amplification grade (Invitrogen) were performed following the manufacturer's instructions. For RT-qPCR was used a SuperScript III ReverseTranscriptase Kit (Invitrogen) and SYBR Green PCR Core Reagents (Applied Biosystems) with an ABI PRISM 7500 instrument (Applied Biosystems) according to the respective manuals. Each mRNA was analyzed in triplicate, normalized to the ribosomal protein S18 content using the comparative Cq method (ΔΔCq). All as previously described (Castillo-Briceño et al., 2009, 2010). The primers used are shown in Supplementary Table 1.

### 2.6 Statistical analysis

The data were analyzed by a two-way analysis of variance (ANOVA) and Bonferroni's multiple comparisons post-test. All the analyses were performed using Prism 5 for Windows version 5.00 in accordance with the appropriate experimental design.

## 3 Results

### 3.1 Mg^2+^-dependent cell attachment specifically differs among collagen-related peptides

**Fig. 1:**
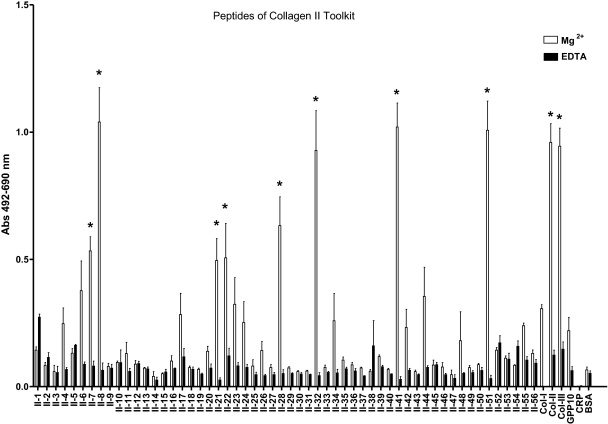
Gilthead seabream fibroblasts (SAF-1) present differential early attachment affinity to COL-II Toolkit peptides. Cells were incubated for 50 min in the presence of 5 mM Mg^2+^ to enhance or 5 mM EDTA to inhibit I-domain and I-like domain mediated cell binding. GXX′GEX″-containing peptides (II-7, -8, -28) and RGD-related peptides (II-8, -21, -22, -32, -41, -51) showed significantly higher adhesion affinity than their respective EDTA and BSA controls (* is according to Bonferroni test from a 2-way ANOVA, *p* < 0.05). Each bar represents the mean ± S.D. of two independent experiments performed in triplicate.

**Fig. 2:**
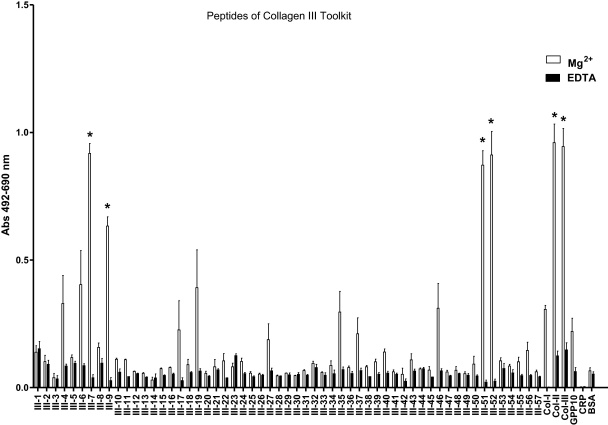
Gilthead seabream fibroblasts (SAF-1) present differential early attachment affinity to COL-III Toolkit peptides. Cells were incubated for 50 min in the presence of 5 mM Mg^2+^ to enhance or 5 mM EDTA to inhibit I-domain and I-like domain mediated cell binding. GXX′GEN-containing peptide (III-7) and RGD-related peptides (III-9, -51, -52) showed significantly higher adhesion affinity than their respective EDTA and BSA controls (* is according to Bonferroni test from a 2-way ANOVA, *p* < 0.05). Each bar represents the mean ± S.D. of two independent experiments performed in triplicate.

**Fig. 3:**
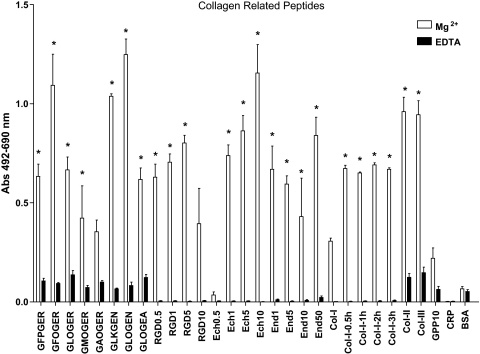
Gilthead seabream fibroblasts (SAF-1) present differential early attachment affinity to specific COL and RGD-related peptides. Cells were incubated for 50 min in the presence of 5 mM Mg^2+^ to enhance or 5 mM EDTA to inhibit I-domain and I-like domain mediated cell binding. Native and denatured (0.5–3 h at 60 °C) collagens, and the COL derived peptides were used at 10 μg/ml. RGD triplet, echistatin (Ech) and endostatin (End) were tested at different concentrations (0.5–50 μg/ml as indicated). Native COL-II and -III, and denatured COL-I, as well as the GXX′GEX″ specific motifs tested (except GAOGER), RGD, Ech and End showed significantly higher adhesion affinity than their respective EDTA and BSA controls (* is according to Bonferroni test from a 2-way ANOVA, *p* < 0.05). Each bar represents the mean ± S.D. of three independent experiments performed in triplicate.

The end-point adhesion assay results showed that SAF-1 cells had higher affinity for GLOGER-, GFOGER- and GLKGENGLOGEN-containing peptides in COL-II (Fig. 1, Supplementary Table 2) and COL-III (Fig. 2, Supplementary Table 3) Toolkits. Other peptides without GXX′GEX″ motifs, but with the RGD sequence, also presented high affinity, e.g., COL-II-51 (Fig. 1), COL-III-51 and COL-III-52 peptides (Fig. 2), all of which contain the sequence GPQGPRGDK (Supplementary Tables 2 and 3). The exceptions were the peptides COL-II-21, COL-II-22 (Supplementary Table 2) and COL-III-9 (Supplementary Table 3) which do not contain any of the mentioned sequences, but presented moderate affinity. Further, the assay with specific collagen-related motifs and fragments shown in Fig. 3 revealed that (i) most of the GXX′GEX″ motifs were able to support cell adhesion, with the highest affinity to GFOGER, GLKGEN and GLOGEN; (ii) only GFOGER and GLOGEN specific motifs showed significantly higher affinity than COL-II and–III; (iii) the RGD triplet showed moderate affinity while echistatin affinity was highly dependent on the coating concentration, being maximal at 10 μg/ml; and (iv) native COL-II and COL-III, but not COL-I, exhibited high affinities, although COL-I in denatured form was able to double its affinity, while endostatin supported moderate attachment.

### 3.2 GFOGER and GLOGEN promote high surface covering by fish fibroblasts *in vitro*

**Fig. 4:**
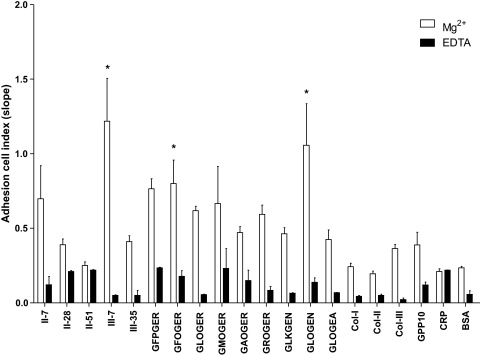
Gilthead seabream fibroblasts (SAF-1) present different surface covering affinity to GLOGEN and GFOGER specific motifs. Incubations were carried out in the presence of 5 mM Mg^2+^ to enhance or 5 mM EDTA to inhibit the I-domain and I-like domain mediated cell binding. Surface covering was continuously read and the slope for the first hour calculated. Only GLOGEN, GFOGER and COL-III-7 spreading affinities were significantly higher than their respective EDTA and BSA controls (* is according to Bonferroni test from a 2-way ANOVA, *p* < 0.05). Each bar represents the mean ± S.D. of three independent experiments performed in triplicate.

In addition, the real-time adhesion assay results (Figs. 4 and 7) showed a delay of about 10 min before the Cell Index began to increase, which may represent the time required for sufficient cells to sediment onto the electrode array. Thereafter, Cell Index rose in approximately linear fashion over the first hour or so, and revealed that *in vitro* the early surface covering by SAF-1 cells for native COL-I, COL-II and COL-III and for COL-II-51 peptide have similar rates than those for GPP10, CRP and BSA controls. Cell Index was also not significantly modified by the peptides containing GXX′GER- sequences which presented low-to-moderate rates. The COL-III-7 peptide which contains GLKGEN and GLOGEN sequences together (Supplementary Table 2) supported the highest covering. In the context of specific collagen motifs, SAF-1 cells only increased Cell Index significantly upon GFOGER and GLOGEN motifs.

### 3.3 Cell adhesion affinity to collagen-derived peptides is strongly modulated after PAMP stimulation

**Fig. 5:**
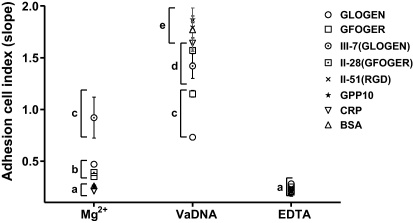
Adhesion affinity of gilthead seabream fibroblasts (SAF-1) is drastically modulated by a pathogen-associated molecular pattern. Incubations were carried out in the presence of 5 mM Mg^2+^ to enhance or 5 mM EDTA to inhibit I-domain and I-like domain mediated cell binding, or 5 mM Mg^2+^ and 50 μg/ml *Vibrio anguillarum* genomic DNA (VaDNA) as a PAMP. Surface covering was continuously read and the slope for the first hour calculated. Mg^2+^ dependent affinity to GXX′GEX″ related peptides slightly increased with VaDNA, but showed lower final levels than reached by the low spreading affinity substrates (COL-II-51, GPP_10_, CRP and BSA). Letters denote significantly different groups, where “a” is equal to EDTA control group (according to Bonferroni test from a 2-way ANOVA, *p* < 0.05). Each dot represents the mean ± S.D. of triplicate samples.

The early cell adhesion affinity for GLOGEN- and GFOGER-containing peptides and specific motifs, measured as the rate of increase of Cell Index during the first hour, showed a slight increase in VaDNA-stimulated cells. However, this increment was minimal compared with the drastic VaDNA induction of cell adhesion to COL-II-51 peptide and GPP10, CRP and BSA controls, which showed the highest rates of covering (Fig. 5).

**Fig. 6:**
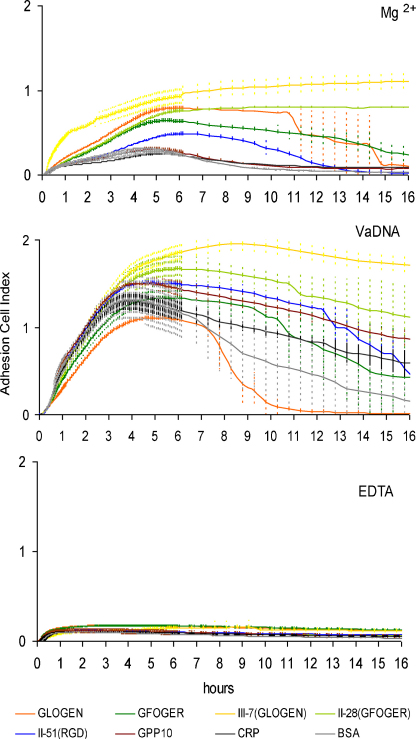
Gilthead seabream fibroblasts (SAF-1) present higher surface covering affinity for GFOGER and GLOGEN motifs in the context of Toolkits. Incubations were carried out in the presence of 5 mM Mg^2+^ to enhance or 5 mM EDTA to inhibit the I-domain and I-like domain mediated cell binding, or 5 mM Mg^2+^ and 50 μg/ml *Vibrio anguillarum* genomic DNA (VaDNA). Surface covering was continuously read during at least 16 h. After maximum covering was reached, cells started to detach except for GLOGEN- and GFOGER-containing peptides. This process was accelerated and enhanced in the presence of VaDNA, where only the GLOGEN-containing peptide (COL-III-7) was able to support cell spreading after 16 h. Each curve represents the mean ± S.D. of triplicate samples.

In the following hours, Mg^2+^ supplemented cells reached the maximum of Cell Index after 5–6 h for all the peptides, while in VaDNA stimulated cells the maximum was reached earlier at 3–4 h (excepting COL-II-28 and COL-III-7 peptides at 4–5 h), doubling the covering reached with Mg^2+^ alone (Fig. 6). After that, cells started detaching from most peptide coatings and particularly fast in VaDNA stimulated cells (especially for GLOGEN motif), except for COL-II-28 and COL-III-7 peptides with Mg^2+^, and only COL-III-7 with VaDNA, which were able to support cell attachment after at least 16 h. In all the cases, EDTA controls presented a very low cell adhesion rate (Fig. 5) and covered surface (Fig. 6).

### 3.4 GFOGER and GLOGEN motifs in suspension increase the mRNA levels of IL-1β and COX-2 in gilthead seabream fibroblasts

**Fig. 7:**
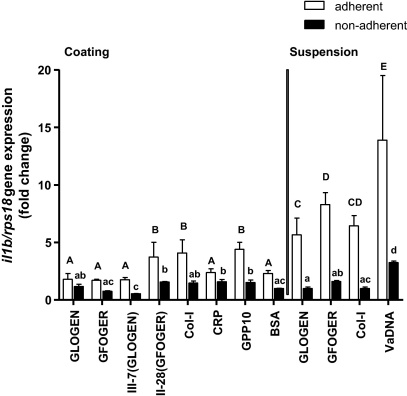
Interleukin-1β (*il1b*) gene expression increases in adherent gilthead seabream fibroblasts stimulated with GFOGER and GLOGEN motifs in suspension. The *il1b* mRNA levels were determined by RT-qPCR in amplification products obtained from SAF-1 cells. Gene expression is normalized against *rps18* and relative to non-adherent cells incubated in BSA-coated plates. Each bar represents the mean ± S.D. of triplicates. Letters denote significantly different groups, where “a” or “A” are not significantly different from the respective non-adherent or adherent BSA controls (according to Bonferroni's post-test for a 2-way ANOVA, *p* < 0.05).

The early adhesion of SAF-1 fibroblasts to any surface, including collagen-derived peptides and BSA, resulted in significant induction of the gene coding for the pro-inflammatory cytokine IL-1β compared to non-adherent cells. Using peptide coatings, there was non-significant difference between them, while in suspension, GFOGER, GLOGEN and COL-I induced a clear increase of IL-1β expression compared to BSA control, but only in cells that became attached to the surface, not to those remaining in suspension. That differs from VaDNA-stimulated cells, which showed a high increase of IL-1β in both adherent and non-adherent cell fractions compared to their correspondent BSA control (Fig. 7). In addition, the expression of cyclooxygenase 2 (COX-2), another pro-inflammatory molecule, showed the same tendency, except for GLOGEN which have non-significant changes even in suspension, while the expression of transforming growth factor β1 (TGFB1), which is an anti-inflammatory cytokine, was non-significantly modulated in all the cases (data not shown).

As regards the ECM-related molecules analyzed (MMP13, COL1A1 and ITGB1a), only ITGB1a showed a notable significant induction in non-adherent cells compared with the adherent cells (data not shown). However, in all the immune- and ECM-related genes analyzed we found that the expression levels were significantly related to the peptides and their synergistic interaction with the adhesion status of the cells (Table 1).

### 3.5 Modeled ITGB1a-GFOGER and -GLOGEN complexes present higher electrostatic stability than ITGB1a-CRP control complex

The modeling analysis of the putative complexes formed between the ITGB1a I-like domain and the GFOGER, GLOGEN or CRP motifs showed that complex models with GFOGER and GLOGEN specific motifs have more favorable electrostatic energies than with CRP control peptide (Table 2), using Mg^2+^ in the active binding site, in agreement with the results obtained in the adhesion assays. This suggests that the sequences may have the same binding mode to the gilthead seabream ITGB1a I-like domain studied here as to the mammalian ITGA2 I-domain, despite their large sequence difference.

## 4 Discussion

Fibroblast adhesion to collagen and other ECM structural components such as fibronectin and laminin has been widely described, and is considered important in morphogenesis, tissue remodeling, wound healing and injury repair processes in mammals, with also a few studies in other vertebrate models. In that sense, despite the limitations of cell lines as experimental models, SAF-1 fibroblasts were very useful to assess the Mg^2+^-dependent affinity of collagen motifs in fish, as previously performed with constitutively adherent cell lines in mammals (Xiong and Zhang, 2001; Siljander et al., 2004; Raynal et al., 2006; Farndale et al., 2008). Further, our results deepened this knowledge in relation to teleost fish fibroblast behavior and allowed us to identify specific collagen motifs involved in the early adhesion of gilthead seabream fibroblasts.

During early fish fibroblast attachment, the generally higher affinities of GXX′GEX″ motif compared with their parent Toolkit peptides suggests that the surrounding sequence is important, as described for GLSGER (COL-III-46) with human platelets (Siljander et al., 2004; Herr and Farndale, 2009). It could also relate to the effect of the peptides 3D structure and the helix conformation as proposed for GFOGER with human fibrosarcoma cells and platelets, and rat Rugli cells (Knight et al., 2000; Herr and Farndale, 2009). These considerations particularly apply to the very high affinity of COL-III-7 peptide, where the high affinity of GLOGEN motif may be improved by the nearby GLKGEN motif, which alone offers a moderate-to-high attachment affinity.

Early attachment affinity levels for GXX′GEX″ specific motifs and the fish fibroblast can be summarized as GLOGEN ≈ GFOGER > GLKGEN > GFPGER ≈ GLOGER ≈ GLOGEA > GMOGER > GAOGER ≈ GPP10 > CRP ≈ BSA, based on the significance (*p* < 0.05) of the differences between groups. This is in broad agreement with the series observed in mammals with GLOGEN and GFOGER as high affinity motifs for platelet integrin α2β1 (Raynal et al., 2006; Farndale et al., 2008; Herr and Farndale, 2009), although GLKGEN was previously found to be at best of low affinity (Raynal et al., 2006). The cause of this variability is unlikely to be phylogenetic, since these GXX′GEX″ sequences and location in collagen are very well conserved in all vertebrates (Siljander et al., 2004; Heino et al., 2009), but could be due to the cellular context and other differences in the receptors involved (Siljander et al., 2004; Munnix et al., 2008).

In addition, Cell Index rates for the above motifs can be summarized as GLOGEN ≈ GFOGER > GFPGER ≈ GLOGER ≈ GMOGER ≈ GAOGER ≈ GROGER ≈ GLKGEN ≈ GLOGEA ≈ GPP10 ≈ COL-III > COL-I ≈ COL-II ≈ CRP ≈ BSA, indicating that GLOGEN and GFOGER are not just able to support early attachment but to facilitate optimum cell spreading in fish fibroblasts. Conversely, the other motifs and their parent Toolkit peptides (including GFOGER-containing peptide, COL-II-28) and the native collagens (COL-I, -II and -III) are able to improve the early attachment but do not modulate the Cell Index and thus the surface covering and cell spreading. This adhesion affinity profile for collagen GXX′GEX″ motifs and SAF-1 cells coincides well with the behavior of human platelets (Siljander et al., 2004; Raynal et al., 2006; Munnix et al., 2008) and fibrosarcoma cell line HT1080 (Farndale et al., 2008).

The adhesion affinity profile for GXX′GEX″-related peptides with the fish fibroblasts, and particularly its high Mg^2+^-dependent affinity for GLOGEN and GFOGER, leads us to suppose that their binding is mediated by ITGB1 heterodimers containing an extracellular I-domain (Perret et al., 2003; Raynal et al., 2006; Farndale et al., 2008; Herr and Farndale, 2009). The receptor involved may be related to mammalian integrin α1β1 (ITGA1:ITGB1), considering its lower affinity for GMOGER and GROGER motifs, which are described as having moderate-high affinity for ITGA2:ITGB1 and ITGA11:ITGB1 in human and rodents (Siljander et al., 2004; Raynal et al., 2006; Farndale et al., 2008; Munnix et al., 2008).

The RGD motif occurs in protruding loops of several integrin-binding proteins, notably fibronectin and fibrinogen, which can bind to the I-like domain of β subunits of α5β1, αIIbβ3 and αVβ3 (Dobler et al., 2006; Raynal et al., 2006; Heino et al., 2009). Although fibrillar collagens contain RGD motifs, for example in the indicated Toolkit peptides, these are constrained in a more rigid, triple-helical conformation, and cannot bind to mammalian integrins unless the collagen becomes denatured (unfolded). In our study, all the Toolkit peptides that contain the RGD motif support early attachment of fish fibroblasts, similar to that of the high affinity GXX′GEX″ peptides. In addition, the non-collagen and non-triple helix peptide echistatin reaches the same affinity level as collagen peptides at the same concentration (10 μg/ml), but the RGD motif alone reaches only low-moderate levels, suggesting that RGD needs to be constrained in a peptide strand to improve its affinity, as suggested for fibronectin RGD (Takagi, 2004; Daley et al., 2008).

It was also remarkable that, in the Toolkits context, COL-II contained more adhesion affinity sites than COL-III for fish fibroblasts, although both parent collagens in the native form showed similar affinity. This is probably due to a cryptic location of the binding affinity sites in the native collagens, which would be recognized by the cell receptors only when exposed (Heino et al., 2009) in denatured forms or, as in this case, in synthetic short peptides. This concept is supported by the increase in COL-I affinity after denaturing.

The high stability of the ITGB1a I-like domain and GFOGER or GLOGEN complex models predicted for gilthead seabream compared with CRP complex suggests that this receptor subunit has a binding affinity similar to that demonstrated for ITGA2, whose binding to GFOGER motif is mediated by its I-domain in a Mg^2+^ dependent manner (Emsley et al., 2000). Hence, it is tempting to speculate that the ITGB1a I-like domain, which is constitutively expressed in gilthead seabream (Castillo-Briceño et al., 2010), can act as an extracellular matrix receptor in fish fibroblasts and also binds GXX′GEX″ motifs. This role would be complementary to ITGA I-domain, whose sequence is well conserved, but its functionality may present some important evolutionary variations, e.g. it is not able to bind to GFOGER and its related motifs in the urochordate *Ciona intestinalis* (Tulla et al., 2007). If this speculation is well founded, this binding mechanism would relate to the ligation of ITGB1 I-like domain to ECM proteins when there is no ITGA I-domain in the integrin heterodimer (Takagi and Springer, 2002; Barczyk et al., 2010). However, it is clear that, directly or indirectly, ITGB1a plays an important part in the initial adhesion of fish fibroblasts, a role that is supported by its higher expression in suspended than in adhered fibroblasts and by the fact that collagen motifs with a high affinity for these cells correspond to the target binding sequences for ITGB1 heterodimers in other vertebrates, as stated for GXX′GEX″ and RGD-containing peptides (Raynal et al., 2006; Farndale et al., 2008; Munnix et al., 2008; Heino et al., 2009). Further experiments aimed at the structural and functional characterization of the recently annotated zebrafish *Danio rerio* ITGA I-domain are required to clarify this important point.

Interestingly, PAMP (VaDNA) stimulation can result in a dramatic generalized induction of the Cell Index rates of fish fibroblasts during early attachment, when the relative affinity levels for the adhesion substrates is reversed to become GPP10 ≈ COL-II-51 (RGD) ≈ BSA ≈ CRP ≥ COL-II-28 (GFOGER) ≈ COL-III-7 (GLOGEN) > GFOGER ≈ GLOGEN. It is easy to imagine that this behavior might constitute a mechanism of primary defence related to wound healing, whereby (i) PAMPs activate the fibroblast integrins so that they are able to bind tightly to lower-affinity substrates and prompt the rapid formation of a layer which acts as a barrier against exogenous agents; and (ii) this early response can be regulated by GFOGER and GLOGEN specific motifs, resulting in fibroblast adhesion levels higher than that in Mg^2+^ conditions but lower than the other substrates during immunological challenge. This role for GXX′GEX″ agrees with the highly studied capacity of collagens and their derived peptides to differentially direct the phases of wound healing or tissue repairing and regeneration processes, such as fibroblasts adhesion and hemostasis (Rothamel et al., 2004; Wolf et al., 2009; Damodarasamy et al., 2010).

Wound healing processes include specific, highly integrated and overlapping phases such as hemostasis, inflammation, proliferation, the formation of granulation tissue, reepithelialization, matrix formation, tissue remodeling or resolution (Guo and Dipietro, 2010), during which the ECM components have a pivotal role (Tran et al., 2004). In this model, it is possible to speculate that SAF-1 cells mimic the general behavior of fibroblasts in response to wound healing, and that the significantly increased expression of IL-1β and COX-2 genes (but not of TGFB1, MMP-13 and COL1A1 genes) after adhesion and during early attachment is part of the inflammatory phase. Interestingly, the more pronounced increase in pro-inflammatory molecules when the collagen peptides are present in suspension, rather than immobilized as substrate, would reflect the described role for collagen as a damage signal in fish (Castillo-Briceño et al., 2009) and its suggested activity as an intermediate signal in the activation of fish phagocytes (Castillo-Briceño et al., 2010). This would be connected with signaling mechanisms of the so-called “defense collagens”, their role in the development of an appropriate immune response and the clearance of pathogens from the organism (Fraser and Tenner, 2008). This effect, at least in the case of fish fibroblasts, seems to be independent of the GXX′GEX″ motifs availability or cell adhesion affinity, since the effect of the GLOGEN or GFOGER motif did not differ from that of native COL-I.

Finally, it may be concluded that SAF-1 cells are a feasible model for studying the adhesive affinity and recognition of collagen peptides in fish. Furthermore, these cells have a Mg^2+^ dependent affinity profile of adhesion very similar to that described for mammalian I- and I-like domain in ITGB1 heterodimers. In addition, GXX′GEX″ motifs are able to differentially modify their behavior and pro-inflammatory gene expression profile, which can be useful for studies in wound healing mechanisms and the involvement of collagen during the early inflammatory response.

## Figures and Tables

**Table 1:** Percentage of gene expression variation related to the COL-derived peptides, cell adhesion and their interactions in SAF-1 cells after 1 h of incubation.

Gene	% of total gene expression variation			*P* value summary (*p* < 0.05)*		
	Peptide	Adhesion	Interaction	Peptide	Adhesion	Interaction
*il1b*	39.70	27.97	21.16	***	***	***
*cox2*	61.20	4.85	33.39	***	***	***
*tgfb1*	42.31	0.02	56.69	***	ns	***
*mmp13*	28.34	25.31	40.83	***	***	***
*col1a*	62.31	1.72	31.58	***	***	***
*itgb1a*	32.04	20.27	46.26	***	***	***

ns = not significant.

* Significant differences were calculated with a two-way ANOVA and Bonferroni post-test.

*** The effect is considered extremely significant.

**Table 2:** Electrostatic stability (free energy of binding) for ITGB1a and COL specific motifs modeled complexes.

Complex	**Δ***G* (energy of complex − energy of separated proteins)
ITGB1a-GFOGER	−86 ± 53 kcal/mol^b^
ITGB1a-GLOGEN	−67 ± 45 kcal/mol^b^
ITGB1a-CRP	−36 ± 37 kcal/mol^a^

Letters denote statistically significant different groups after a 1-way ANOVA and Bonferroni post-test (*p* < 0.05).
