# Supplementary material for: A role for specific collagen motifs during wound healing and inflammatory response of fibroblasts in the teleost fish gilthead seabream
Source: Mol Immunol. 2011 Mar;48(6-7):826–34. doi: 10.1016/j.molimm.2010.12.004 (PMC3048961; doi:10.1016/j.molimm.2010.12.004)
Supplement: Supplementary file 1 [file mmc1.doc]

**Supplementary Table 1.** Primer sequences used for gene expression analysis. The gene symbols follow the Zebrafish Nomenclature Guidelines (<http://zfin.org/zf info/nomen.html>).

| ***Gene*** | **Accession number** | **Primer name** | **Sequence (5’→3’)** |
| --- | --- | --- | --- |
| *rps18* | AM490061 | F | AGGGTGTTGGCAGACGTTAC |
|  |  | R | CTTCTGCCTGTTGAGGAACC |
| *il1b* | AJ277166 | F3 | ATGCCCGAGGGGCTGGGC |
|  |  | R2 | CAGTTGCTGAAGGGAACAGAC |
| *cox2* | AM296029 | F1 | GAGTACTGGAAGCCGAGCAC |
|  |  | R1 | GATATCACTGCCGCCTGAGT |
| *tgfb1* | AF424703 | F | AGAGACGGGCAGTAAAGAA |
|  |  | R | GCCTGAGGAGACTCTGTTGG |
| *mmp13* | AM905935 | F | CGGTGATTCCTACCCATTTG |
|  |  | R | TGAGCGGAAAGTGAAGGTCT |
| *col1a1* | DQ324363 | F2 | GCTCTCAGCCAGAGGATGTC |
|  |  | R2 | TGTAGGCGATGCTGTTCTTG |
| *itgb1a* | FN649420 | F2 | AAGGGAGACGAGTTCAATCGGT |
|  |  | R1 | CACCAGCAGACGAGTCACAT |
