# Supplementary material for: A role for specific collagen motifs during wound healing and inflammatory response of fibroblasts in the teleost fish gilthead seabream
Source: Mol Immunol. 2011 Mar;48(6-7):826–34. doi: 10.1016/j.molimm.2010.12.004 (PMC3048961; doi:10.1016/j.molimm.2010.12.004)
Supplement: Supplementary file 2 [file mmc2.doc]

**Supplementary Table 2.** Sequences of Collagen type II Toolkit Peptides

| **Peptide** | **Specific Sequence** |
| --- | --- |
| 1 | GPMGPMGPRGPOGPAGAOGPQGFQGNO |
| 2 | GPQGFQGNOGEOGEOGVSGPMGPRGPO |
| 3 | GPMGPRGPOGPOGKOGDDGEAGKOGKA |
| 4 | GEAGKOGKAGERGPOGPQGARGFOGTO |
| 5 | GARGFOGTOGLOGVKGHRGYOGLDGAK |
| 6 | GYOGLDGAKGEAGAOGVKGES**GSOGEN** |
| 7 | GES**GSOGEN**GSOGPMGPR**GLOGER**GRT |
| 8 | **GLOGER**GRTGPAGAA**GA*RGN*D**GQOGPA |
| 9 | GNDGQOGPAGPOGPVGPAGGOGFOGAO |
| 10 | GGOGFOGAOGAKGEAGPTGARGPEGAQ |
| 11 | GARGPEGAQGPRGEOGTOGSOGPAGAS |
| 12 | GSOGPAGASGNOGTDGIOGAKGSAGAO |
| 13 | GAKGSAGAOGIAGAOGFOGPRGPOGPQ |
| 14 | GPRGPOGPQGATGPLGPKGQTGEOGIA |
| 15 | GQTGEOGIAGFKGEQGPKGEOGPAGPQ |
| 16 | GEOGPAGPQGAOGPAGEEGKRGARGEO |
| 17 | GKRGARGEOGGVGPIGPOGERGAOGNR |
| 18 | GERGAOGNRGFOGQDGLAGPK**GAOGER** |
| 19 | GPK**GAOGER**GPSGLAGPKGANGDOGRO |
| 20 | GANGDOGROGEOGLOGARGLTGROGDA |
| 21 | GLTGROGDAGPQGKVGPSGAOGE***DGR***O |
| 22 | GAOGE***DGR***OGPOGPQGARGQO**GVMGFO** |
| 23 | GQO**GVMGFO**GPKGANGEOGKAGEKGLO |
| 24 | GKAGEKGLOGAOGLRGLOGKDGETGAA |
| 25 | GKDGETGAAGPOGPAGPAGERGEQGAO |
| 26 | GERGEQGAOGPSGFQGLOGPOGPOGEG |
| 27 | GPOGPOGEGGKOGDQGVOGEAGAOGLV |
| 28 | GEAGAOGLVGPRGER**GFOGER**GSOGAQ |
| 29 | GERGSOGAQGLQGPRGLOGTOGTDGPK |
| 30 | GTOGTDGPKGASGPAGPOGAQGPOGLQ |
| 31 | GAQGPOGLQ**GMOGER**GAAGIAGPKGDR |
| 32 | GIAGPKGD***RGD***VGEKGPEGAOGKDGGR |
| 33 | GAOGKDGGRGLTGPIGPOGPAGANGEK |
| 34 | GPAGANGEKGEVGPOGPAGSAGARGAO |
| 35 | GSAGAR**GAOGER**GETGPOGPAGFAGPO |
| 36 | GPAGFAGPOGADGQOGAKGEQGEAGQK |
| 37 | GEQGEAGQKGDAGAOGPQGPSGAOGPQ |
| 38 | GPSGAOGPQGPTGVTGPKGARGAQGPO |
| 39 | GARGAQGPOGATGFOGAAGRVGPOGSN |
| 40 | GRVGPOGSNGNOGPOGPOGPSGKDGPK |
| 41 | GPSGKDGPKGA***RGD***SGPOGRAGEOGLQ |
| 42 | GRAGEOGLQGPAGPOGEKGEOGDDGPS |
| 43 | GEOGDDGPSGAEGPOGPQGLAGQRGIV |
| 44 | GLAGQRGIVGLOGQRGERGFOGLOGPS |
| 45 | GFOGLOGPSGEOGKQGAOGASGDRGPO |
| 46 | GASGDRGPOGPVGPOGLTGPAGEOGRE |
| 47 | GPAGEOGREGSOGADGPOGRDGAAGVK |
| 48 | GRDGAAGVKGDRGETGAVGAOGAOGPO |
| 49 | GAOGAOGPOGSOGPAGPTGKQGDRGEA |
| 50 | GKQGDRGEAGAQGPMGPSGPAGARGIQ |
| 51 | GPAGARGIQGPQGP***RGD***KGEAGEOGER |
| 52 | GEAGEOGERGLKGHRGFTGLQGLOGPO |
| 53 | GLQGLOGPOGPSGDQGASGPAGPSGPR |
| 54 | GPAGPSGPRGPOGPVGPSGKDGANGIO |
| 55 | GKDGANGIOGPIGPOGPRGRSGETGPA |
| 56 | GPRGRSGETGPAGPOGNOGPOGPOGPO |

Candidate sequences for SAF-1 adhesion affinity are highlighted: **GXX’GEX”** motifs, ***RGD*** related peptides, Sequences selected from residues overlapping analysis in peptides with significant affinity, **other non-GXX’GEX”** possible candidates.
