# Supplementary material for: A role for specific collagen motifs during wound healing and inflammatory response of fibroblasts in the teleost fish gilthead seabream
Source: Mol Immunol. 2011 Mar;48(6-7):826–34. doi: 10.1016/j.molimm.2010.12.004 (PMC3048961; doi:10.1016/j.molimm.2010.12.004)
Supplement: Supplementary file 3 [file mmc3.doc]

**Supplementary Table 3.** Sequences of Collagen type III Toolkit Peptides

| **Peptide** | **Specific Sequence** |
| --- | --- |
| 1 | GLAGYOGPAGPOGPOGPOGTSGHOGSO |
| 2 | GTSGHOGSOGSOGYQGPOGEOGQAGPS |
| 3 | GEOGQAGPSGPOGPOGAIGPSGPAGKD |
| 4 | GPSGPAGKDGESGRO**GROGER**GLOGPO |
| 5 | GERGLOGPOGIKGPAGIOGFOGMKGHR |
| 6 | GFOGMKGHRGF***DGR***NGEKGETGAOGLK |
| 7 | GETGAO**GLKGENGLOGEN**GAOGPMGPR |
| 8 | GAOGPMGPR**GAOGER**GROGLOGAAGAR |
| 9 | GLOGAA**GA*RGN*D**GARGSDGQOGPOGPO |
| 10 | GQOGPOGPOGTAGFOGSOGAKGEVGPA |
| 11 | GAKGEVGPAGSOGSNGAOGQRGEOGPQ |
| 12 | GQRGEOGPQGHAGAQGPOGPOGINGSO |
| 13 | GPOGINGSOGGKGEMGPAGIOGAOGLM |
| 14 | GIOGAOGLMGARGPOGPAGANGAOGLR |
| 15 | GANGAOGLRGGAGEOGKNGAKGEOGPR |
| 16 | GAKGEOGPRGERGEAGIOGVOGAKGED |
| 17 | GVOGAKGEDGKDGSOGEOGANGLOGAA |
| 18 | GANGLOGAAGERGAOGFRGPAGPNGIO |
| 19 | GPAGPNGIOGEKGPAGERGAOGPAGPR |
| 20 | GAOGPAGPRGAAGEOGRDGVOGGOGMR |
| 21 | GVOGGOGMRGMOGSOGGOGSDGKOGPO |
| 22 | GSDGKOGPOGSQGESGROGPOGPSGPR |
| 23 | GPOGPSGPRGQO**GVMGFO**GPKGNDGAO |
| 24 | GPKGNDGAOGKNGERGGOGGOGPQGPO |
| 25 | GGOGPQGPOGKNGETGPQGPOGPTGPG |
| 26 | GPOGPTGPGGDKGDTGPOGPQGLQGLO |
| 27 | GPQGLQGLOGTGGPOGENGKOGEOGPK |
| 28 | GKOGEOGPKGDAGAOGAOGGKGDAGAO |
| 29 | GGKGDA**GAOGER**GPOGLAGAOGLRGGA |
| 30 | GAOGLRGGAGPOGPEGGKGAAGPOGPO |
| 31 | GAAGPOGPOGAAGTOGLQ**GMOGER**GGL |
| 32 | **GMOGER**GGLGSOGPKGDKGEOGGOGAD |
| 33 | GEOGGOGADGVOGKDGPRGPTGPIGPO |
| 34 | GPTGPIGPOGPAGQOGDKGEGGAOGLO |
| 35 | GEGGAOGLOGIAGPRGSOGERGETGPO |
| 36 | GERGETGPOGPAGFOGAOGQNGEOGGK |
| 37 | GQNGEOGGKGERGAOGEKGEGGPOGVA |
| 38 | GEGGPOGVAGPOGGSGPAGPOGPQGVK |
| 39 | GPOGPQGVKGERGSOGGOGAAGFOGAR |
| 40 | GAAGFOGARGLOGPOGSNGNOGPOGPS |
| 41 | GNOGPOGPSGSOGKDGPOGPAGNTGAO |
| 42 | GPAGNTGAOGSOGVSGPKGDAGQOGEK |
| 43 | GDAGQOGEKGSOGAQGPOGAOGPLGIA |
| 44 | GAOGPLGIAGITGARGLAGPOGMOGPR |
| 45 | GPOGMOGPRGSOGPQGVKGESGKOGAN |
| 46 | GESGKOGAN**GLSGER**GPOGPQGLOGLA |
| 47 | GPQGLOGLAGTAGEOGRDGNOGSDGLO |
| 48 | GNOGSDGLOGRDGSOGGKGDRGENGSO |
| 49 | GDRGENGSOGAOGAOGHOGPOGPVGPA |
| 50 | GPOGPVGPAGKSGDRGESGPAGPAGAO |
| 51 | GPAGPAGAOGPAGSRGAOGPQGP***RGD***K |
| 52 | GPQGP***RGD***KGETGERGAAGIKGHRGFO |
| 53 | GIKGHRGFOGNOGAOGSOGPAGQQGAI |
| 54 | GPAGQQGAIGSOGPAGPRGPVGPSGPO |
| 55 | GPVGPSGPOGKDGTSGHOGPIGPOGPR |
| 56 | GPIGPOGPRGNRGERGSEGSOGHOGQO |
| 57 | GERGSEGSOGHOGQOGPOGPOGAOGPC |

Candidate sequences for SAF-1 adhesion affinity are highlighted: **GXX’GEX”** motifs, ***RGD*** related peptides, Sequences selected from residues overlapping analysis in peptides with significant affinity, **other non-GXX’GEX”** possible candidates.
